# Metabolomic profiling of infants undergoing cardiopulmonary bypass and association with clinical outcomes: a systematic review

**DOI:** 10.3389/fcvm.2024.1491046

**Published:** 2024-11-14

**Authors:** Leonardo Meggiolaro, Laura Moschino, Matteo Stocchero, Giuseppe Giordano, Vladimiro Vida, Giovanni Di Salvo, Eugenio Baraldi

**Affiliations:** ^1^ Neonatal Intensive Care Unit, Department of Women’s and Children’s Health, University of Padova, Padova, Italy; ^2^ Fondazione Istituto di Ricerca Pediatrica Città Della Speranza, Padova, Italy; ^3^ Paediatric Cardiac Surgery, Padova University Hospital, Padova, Italy; ^4^ Paediatric Cardiology Unit, Department of Women’s and Children’s Health, Padova University Hospital, Padova, Italy

**Keywords:** metabolomics, heart defects, congenital, clinical outcomes, bypass, cardiopulmonary, infants, newborn

## Abstract

**Introduction:**

The incidence of adverse short-term outcomes for infants who undergo complex congenital heart disease (CHD) surgery with cardiopulmonary bypass (CPB) is still high. Early identification and treatment of high-risk patients remain challenging, especially because clinical risk factors often fail to explain the different outcomes of this vulnerable population. Metabolomics offers insight into the phenotype of the patient and the complex interplay between the genetic substrate and the environmental influences at the time of sampling. For these reasons, it may be helpful to identify the mechanisms of physio-pathological disruptions experienced in neonates undergoing congenital heart surgery and to identify potential therapeutic targets.

**Methods:**

We conducted a systematic review (*PROSPERO*: ID 565112) of studies investigating the association between targeted or untargeted metabolomic analysis of infants undergoing elective surgery with CPB for CHD and clinical outcomes. The PRISMA guidelines were followed. We searched MEDLINE via PubMed, EMBASE via Ovid, the Cochrane Central Register of Controlled Trials, the Cochrane Library, ClinicalTrials.gov and the World Health Organization's International Trials Registry and Platform.

**Results:**

Seven studies involving 509 children (aged 1 day to 21.3 months), all of whom underwent cardiac surgery requiring CPB, were included for qualitative analysis. We found associations between metabolomic profiles and various clinical outcomes, such as mortality, acute kidney injury (AKI), and neurological outcomes. Specific metabolites (mainly amino acids, their metabolic products and fatty acids) were identified as potential biomarkers for these outcomes, demonstrating the utility of metabolomics in predicting certain postoperative complications.

**Conclusion:**

The quality of the evidence was limited due to heterogeneity in study designs and small sample sizes, but the findings are promising and suggest that further research is warranted to confirm these associations.

**Systematic Review Registration:**

https://www.crd.york.ac.uk/prospero/, PROSPERO ID 565112.

## 1 Introduction

Congenital heart disease (CHD) represents the most common birth anomaly, with a prevalence of approximately 8:1000 newborns born alive (1). Despite significant improvements in morbidity and mortality over the past 30 years, congenital heart surgery remains a high risk, especially for neonates (1, 2). Current risk models incorporate clinical factors that are associated with increased morbidity and mortality, such as age, weight, preoperative status, comorbidities and surgical complexity. However, clinical risk factors alone often fail to explain the different outcomes of this vulnerable population (3).

Metabolomics is the high-throughput analysis of the metabolome, which is the collection of small molecules within a biological sample, such as cells, tissues, or biological fluids, and includes the intermediate products of metabolic reactions that are required for cells to function (4). This dynamic field of science offers the potential to identify unique metabolic patterns and uncover pathogenetic pathways that may be involved during and after cardiac heart surgery (4, 5). Metabolic profiling has already shown excellent sensitivity and specificity in detecting CHD during fetal life (6). In adult patients with heart failure, metabolic profiling has been shown to provide robust diagnostic and prognostic value in addition to BNP and traditional risk factors [such as for example Left Ventricular Ejection Fraction (LVEF), blood pressure, metabolic syndrome and chronic kidney disease] (7). In bicuspid aortic valve disease, mitral valve disease, rheumatic heart disease, and degenerative aortic valve stenosis, several metabolic biomarkers (such as for example difference in arginine, proline and arachidonic acid metabolism) have been reported to predict patient outcomes after valve repair or replacement (8). Currently, several studies are highlighting the possible role of metabolomics as a powerful tool also to define postoperative metabolic derangements in infants and children with CHD. This technique, applied through a targeted or an untargeted approach, could establish early metabolic biomarkers of postoperative morbidity and mortality and predict outcomes after cardiac surgery, particularly in higher-risk infants (9).

However, studies focusing only on the neonatal population are scarce. We thus decided to conduct a systematic review of studies investigating the association between targeted or untargeted metabolomic profiles of infants undergoing elective surgery with CPB for CHD and clinical outcomes.

## 2 Materials and methods

This review was conducted according to the Preferred Reporting Items for Systematic Reviews and Metanalysis (PRISMA) statement (10).

### 2.1 Inclusion and exclusion criteria

We included randomized, quasi-randomized trials and cohort studies applying targeted or untargeted metabolomics for the prediction of postcardiac surgery outcomes. We excluded case reports, case series, conference abstracts, unpublished studies or studies published in a language other than English or whose full-text was not available. The population was characterized by infants and children (< 18 years of age) who underwent elective cardiac surgery for congenital heart disease and whose blood or urine samples were analysed through targeted or untargeted metabolic profiling obtained pre- and postoperatively. We excluded studies including adults.

The outcomes of interest were mainly short term and included: procedural mortality (all-cause mortality within 30 days or index procedure hospitalization if the postoperative stay is longer than 30 days), the need for extracorporeal membrane oxygenation, cardiac arrest, hypoxemia, acute kidney injury, hepatic injury, the number of PICU-free days at 28 days, the Pediatric Logistic Organ Dysfunction (PELOD) score, the RACHS-1 score, the Inotrope score, the duration of ventilation, the ICU length of stay (LOS), neurodevelopmental assessment and their correlation with metabolic pathways and metabolites.

### 2.2 Research strategy

The electronic search was conducted using MEDLINE via PubMed (up to 31 May 2024), EMBASE via Ovid (up to 31 May 2024), the Cochrane Central Register of Controlled Trials, and the Cochrane Library. We also searched clinical trial databases—ClinicalTrials.gov and the World Health Organization's International Trials Registry and Platform—for ongoing or recently completed trials on 31 May 2024.

All searches were conducted using the following MeSH terms: (metabolomic OR metabolomics) AND (“congenital heart disease” OR “congenital heart defect” OR “heart disease”) AND (infants OR newborn OR neonates)

The search was last updated on 31 May 2024.

### 2.3 Data extraction

Two authors independently conducted the search and identified the records for full-text screening. Conflicts were resolved by consensus or after consultation with a third author. Screening and eligibility assessments were performed using COVIDENCE (http://www.covidence.org/).

## 3 Results

### 3.1 Research strategy

Fifty-one studies were identified through the database search. After applying the exclusion criteria and duplicates removal, 7 studies were included for the purpose of the systematic review.

**Figure 1:**
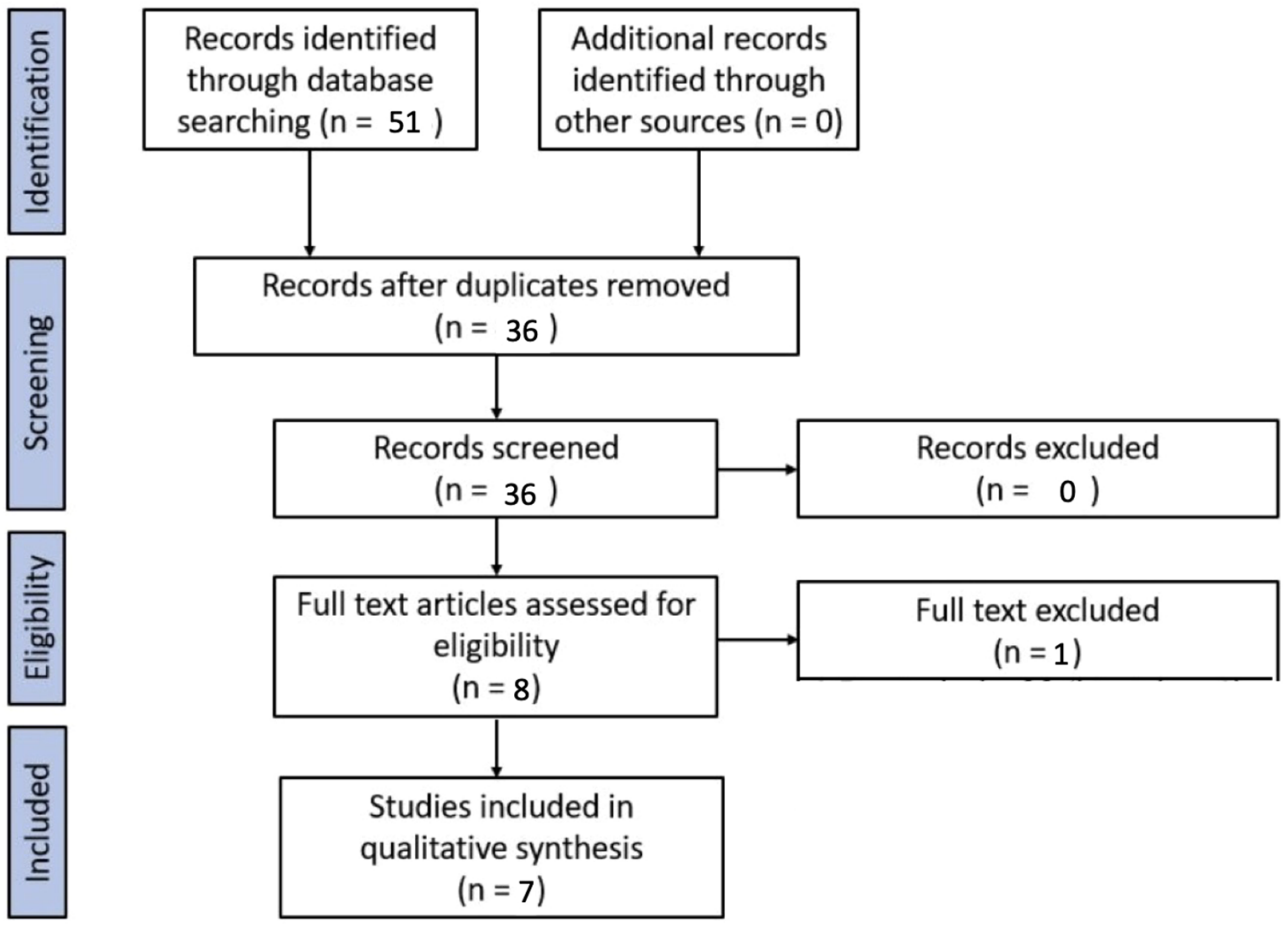
PRISMA flowchart.

A PRISMA flow chart of the screening and selection results is shown in Figure 1. Studies were evaluated for qualitative analysis, but a meta-analysis was not possible due to the heterogeneity of the studies (in particular, different inclusion criteria for the population under study and different outcomes). One study was excluded because, among its exclusion criteria, it included children who had undergone cardiopulmonary bypass (11).

### 3.2 Characteristics of selected studies

The details of the included studies are summarized in Table 1.

**Table 1:** Details of the included studies.

First author, year	Place, time	Sample size; mean age and weight at recruitment; mean age at surgery	Type of CHD (%)	Type of approaches; biological sample (if plasma or serum: P—peripherallyobtained; C—centrally obtained); type of analysis	Principal outcomes (timing of their evaluation)
Goncalo D. S. Correia, 2015	Royal Brompton Hospital	28; 6.6 months (4.0– 18.9), and median weight of 6.2 kg (4.0–8.52); not specified	TOF (28.57); VSD (32.14); HLHS (7.14); AVD (3.57); TA (7.14); TGA (10.71); MVS (3.57); TAPVC (3.57); PA (3.57)	Untargeted; Plasma (P); H-NMR	PICU-free days at 28 days (28 days); Pediatric Logistic Organ Dysfunction (PELOD) score; RACHS-1 score; Inotrope Score; Duration of ventilation (not further specified)
Jesse A. Davidson, 2018	Not specified	82; not specified and median weight of 3.5 Kg (2.1–7.9); 14.5 days (1.0–120)	Specificied only for “non survivors group": Pulmonary atresia/VSD, TAPVR; Tracheal stenosis, VSD, ASD; DORV, ASD; DORV, mitral atresia; HLHS, VSD, PAPVR; D-TGA, aortic arch hypoplasia)	Targeted; Serum (C); MS	Cardiac arrest, need for mechanical circulatory support (extracorporeal membrane oxygenation), death in hospital or death (within 30 days of surgery—inpatient or outpatient), ICU LOS
Vedovelli, 2019	Pediatric Cardiovascular Surgery Unit, “V. Gallucci” center, Padova University Hospital	28; 3.0 months (0.4–6.5) and median weight of 4.8 Kg (3.4–6.6); 3.0 months (0.4–6.5)	TGA (6); TOF (11); Septal Defects (3); Univentricular (8)	Untargeted; Urine; H-NMR	Neuropsychological examinations and an evaluation of the adaptive functioning (e.g., Raven Colored Matrices, NEPSY-II, Coding test of the WISC-IV, visual-motor integration, VABS-I) (at least 18 months after surgery)
Jesse A. Davidson, 2021	Not specified	57; not specified; not specified	Not specified	Targeted; Serum (C); LC-MS/MS	AKI using the Kidney Disease Improving Global Outcomes (KDIGO) criteria with the neonatal modification for subjects ≤28 days of age (at 72 h and 7 days after surgery)
Heibel, 2022	Two North American centers; 2012–2016	149; 8.7 days ± 5.3; not specified; not specified	Biventricular with arch hypoplasia 27 (18); TGA 37 (25); All other biventricular disorders 40 (27); SV 45 (30)	Targeted; Plasma (not specified); MS	Composite morbidity/mortality (met if any of the following occurred after surgery but before hospital discharge: death, extracorporeal membrane oxygenation, cardiac arrest, acute kidney injury, hepatic injury, increasing lactate levels); Cardiac composite (met if any of the following occurred after surgery but before hospital discharge: extracorporeal membrane oxygenation, cardiac arrest or increase in lactate level); hepatic injury and acute kidney injury (after surgery but before discharge)
S.Frank, 2023	Children's Hospital Colorado; not specified	52; 0.348 years (0.123–1.78) and median weight of 5.60 Kg (4.20, 58.0); not specified	Single Ventricle Heart Disease (Ventricle morphology: 10 LV and 42 RV)	Targeted; Serum (C); MS	Hypoxemia defined as an oxygen saturation below 70% (in the first 48 h post intervention); hemodynamic measurements obtained during pre-Stage 2 catheterization; mean saturation levels; endotracheal intubation time; chest tube days; volume of chest tube drainage (post-surgery, not further specified)
S.Frank, 2024	Children's Hospital Colorado; not specified	75; 4.64 months (2.79–13.22) and median weight of 5.70 Kg (3.80–10.40); not specified	Single ventricle heart disease (Ventricle morphology: LV 18)	Targeted; Serum (C); MS	Hypoxemia in the first 48 h post intervention defined as an oxygen saturation below 70% (in the first 48 h post intervention); post- operative length of stay (LOS); need for targeted pulmonary hypertension therapy (at discharge)

In 2015, Correia et al. (5) performed a prospective study on 28 children with CHD who underwent cardiac surgery. The average age of the included patients was 6.6 months ((4.0–18.9) The diagnoses included (n) Tetralogy of Fallot (TOF) (8); Ventricular septal defect (VSD) (9); Hypoplastic left heart syndrome (HLHS) (2); Atrioventricular septal defect (AVD) (1); Truncus arteriosus (TA) (2); Transposition of the great arteries (TGA) (3); Mitral valve stenosis (MVS) (1); Total anomalous pulmonary venous connection (TAPVC) (1); Pulmonary atresia (PA) (1). The study was designed so that after cardiac surgery, a group of patients would be treated by observing tighter glycemic control, while a group would be treated as the standard of care. Peripheral blood was obtained from intravascular lines both preoperatively (at induction of anaesthesia) and at serial time points postoperatively following admission to the PICU: at admission and then at 6, 24 and 48 h post-surgery. Untargeted metabolomic analysis based on Proton Nuclear Magnetic Resonance (H-NMR) of plasma samples was then used to compare the two groups based on the hypothesis that tighter postoperative glycemic control could reduce inflammation and metabolic dysregulation. The study was a pilot exploratory study for hypothesis generation. Data analysis was mainly based on Principle Component Analysis (PCA) and Partial Least Square (PLS)-methods. The experimental design was not explicitly considered in data modelling. Indeed, even if NMR signals of drugs and those related of feed type were removed by data pre-treatment, clinical data were not explicitly used in data modelling to reduced potential confounding effects. Model optimization and validation were not adequately described. The findings should be considered qualitative.

In 2018 and 2021, Davidson et al. (12, 13) organized two secondary analyses from a previously published cohort study that evaluated the role of alkaline phosphatase as a biomarker of postoperative outcomes following neonatal or infant congenital heart disease surgery. The study enrolled infants ≤120 days of age who were scheduled to undergo cardiothoracic surgery with CPB. The studies did not include a control group. Serum samples were obtained preoperatively, during rewarming (before separation from CPB), and 24 h after the patients returned to the ICU. In 2021 a targeted Mass-Spectrometry based analysis was performed if a residual frozen serum sample was still available so that, in 2018, 82 infants were included (not all the infants enrolled in the parent cohort underwent metabolomic profiling) while in 2021, only 57.

At the time of the first study, the average age of the included patients at surgery was 14.5 days (1.0–120), and their average weight at recruitment was 3.5 kg (2.1–7.9). The population was subdivided into a “survivor group” and a “non-survivor group”. Six patients were included in this last group who had the following diagnoses: pulmonary atresia/VSD (Ventricular Septal Defect), TAPVR (Total Anomalous Pulmonary Venous Return); tracheal stenosis, VSD, ASD (Atrial Septal Defect); DORV (Double Outlet Right Ventricle), ASD; DORV, mitral atresia; HLHS (Hypoplastic Left Heart Syndrome), VSD, PAPVR (Partial Anomalous Pulmonary Venous Return); and D-TGA (D-Transposition of the Great Vessels), aortic arch hypoplasia. The outcomes of cardiac arrest, the need for mechanical circulatory support (Extra Corporeal Membrane Oxygenation), death in the hospital or death within 30 days of surgery (inpatient or outpatient) and the ICU LOS were analysed.

The experimental design and the aim were well described. A preliminary investigation highlighted the need of a subject stratification by age. PLS-Discriminant Analysis (DA) and pathway analysis were applied. The experimental design, that considers all the known factors that may influence the serum metabolome, was not used explicitly in data modelling, but single subproblems were solved. The findings should be considered only for hypothesis generation.

At the time of the second analysis, in 2021, the average age and weight of the included patients was not specified but the authors declared that there were no significant clinical differences between the full parent cohort and the metabolomics sub-cohort in terms of baseline and demographic data. The list of diagnoses of patients included in the second study was not specified by the authors. The main objective of the study was to determine whether the perioperative metabolic profiles would discriminate between patients with or without AKI and whether patients with more severe AKI would demonstrate greater changes in their circulating metabolome. Among the patients analysed, 34% had AKI (23% with KDIGO stage 1 AKI and 11% with KDIGO stage 2/3 AKI), and 66% of the infants did not have AKI.

The experimental design and the aim of the study were well described. PLS-DA, pathway analysis and *t*-test controlling the false discovery rate were applied. A PLS-DA model was reported and used for explaining the data variation. The study should be considered an exploratory study.

In 2019, Vedovelli et al. (14) conducted a prospective, observational, single-center study that included 28 children with complex CHD who required CPB during surgery. The mean age of the patients at recruitment was 3.0 months (0.4–6.5), while the average weight was 4.8 kg (3.4–6.6). The study did not include a control group. The diagnoses included (n) TGA (6), TOF (11), septal defects (not further specified if interatrial or interventricular defects) (3), and single ventricle heart disease (8). A urinary catheter was placed after the induction of anaesthesia in the operating room to collect a sample from the urinary reservoir before cardiac surgery. The authors explored whether the untargeted metabolic profile of urine samples based on H-NMR spectroscopy could be a biomarker of impaired neurological outcomes. Based on the metabolomic profile only, patients were classified into 2 different groups: “RED” and “BLACK”. At least 18 months after surgery, neuropsychological examinations were performed to determine whether metabolic profiles could reflect the physiological status before surgery and predict clinical and neurodevelopmental outcomes. The evaluation included the Raven Coloured Matrices, the NEPSY-II, the Coding test of the WISC-IV, the visual-motor integration test, and the Vineland Adaptive Behavior Scales (VABS-I) for adaptive functioning.

Despite the good description of the experimental design and the aims, the data analysis was potentially not adequate to support the findings, even if clear trends seemed to suggest a relationship between the urinary metabolome and neurodevelopmental outcomes. Indeed, the authors claimed the presence of 2 groups of patients only based on the visual inspection of the PCA score scatter plot without proving the reliability of the groups by statistical tools. Moreover, even if PLS-DA has been cited as techniques for data analysis, no PLS models were presented and univariate data analysis, that may lead to new insights into the collected data, was not performed.

More recently, in 2022, Heibel et al. (15) carried out a secondary analysis of a multicenter, double-blind, randomized, placebo-controlled trial (Corticosteroid Therapy in Neonates Undergoing Cardiopulmonary Bypass trial; ClinicalTrials.gov Identifier: NCT01579513). The study included 149 neonates (≤ 1 month) scheduled to undergo cardiac surgery. The average age at the time of surgery was 8.7 ± 5.3 days, while the mean weight was not specified. The study did not include a control group. The diagnoses included the following (n): biventricular heart with arch hypoplasia (18); TGA (25); all other biventricular disorders (27); and single ventricle heart disease (30). Plasma samples were collected before skin incision, immediately after CPB and 12 h after surgery. Targeted metabolite levels were determined by high-resolution tandem liquid chromatography and Mass Spectrometry. PCA and regression analysis were used to assess associations between metabolic profiles and outcomes, with the generation of two types of models: a base clinical model and a base model + metabolites (namely, principal components PC1-6). Among the outcomes for which an association with metabolic profiles was explored were death, renal dysfunction, hepatic dysfunction and cardiac arrest. The composite morbidity/mortality was met if any of the following occurred after surgery but before hospital discharge: death, extracorporeal membrane oxygenation, cardiac arrest, acute kidney injury, hepatic injury and/or increasing lactate levels.

The experimental design and the aim of the study were well described. The strategy for data analysis composed of several steps was very complex logistic-regression, PCA combined with logistic-regression and variable selection, and variable selection and resampling were used in a 3-step data analysis strategy. The strategy may be adequate in principle, but it is not clear how over-fitting and false discovery was controlled or taken into account. In 2023 and 2024, S. Frank (16, 17) conducted two prospective cohort studies on infants aged 1 month to 2 years with Single-Ventricle Heart Disease (SVHD) who were undergoing pre-stage 2 cardiac catheterization or stage 2 surgical palliation without a catheterization plan. Both studies applied a targeted analysis using serum samples and included a control group. First, 52 infants were included in the study. The mean age was 0.348 years (0.123–1.78) and median weight was 5.60 Kg (4.20, 58.0). The objective was to evaluate whether the circulating metabolome and metabolite levels were associated with pulmonary vascular inadequacy. Two systemic venous and pulmonary venous samples were obtained under general anesthesia during cardiac catheterization or surgery and 2 h post-operation. Their targeted profiles were then compared to a similar-aged healthy control group (*n* = 48) composed by subjects from the surgical schedule at Children's Hospital Colorado to evaluate whether these patterns could provide insights into pulmonary vascular readiness and postoperative complications related to insufficient pulmonary blood flow. Control subject inclusion criteria included weight >4 kg and age 3 to 12 months. All controls were undergoing anesthesia for an elective, noncardiac procedure with a clinical need for intravenous access.

The primary outcomes were hypoxemia burden in the first 48 postoperative hours and postoperative length of stay. Secondary outcomes were hemodynamic measurements obtained during pre-Stage 2 catheterization, mean saturation levels, endotracheal intubation time and chest tube days.

The experimental design and the aim of the study were well described. The analysis of the clinical data discovered a potential bias due to age, but this bias was not considered in data modelling. Random Forest, pathway analysis and linear regression models controlling false discovery rate were applied. The study was exploratory.

In the second study, 75 infants were included. The mean age was 4.64 months (2.79–13.22). The purpose of this study was to identify changes in arginine and NO metabolites associated with morbidity in infants with SVHD and to understand their potential role in predicting postoperative complications and guiding targeted therapies. The methods of collecting biological samples followed the same procedures as in the previous study. Also in this case, the targeted profiles were compared with a control group (*n* = 50) recruited in the same way as for the prior study. The primary outcomes were hypoxemia burden in the first 48 postoperative hours and postoperative length of stay, while the secondary outcomes were hemodynamic measurements obtained during pre-Stage 2 catheterization and the need for targeted pulmonary hypertension therapy at discharge.

The study was hypothesis-driven, and the experimental design was well described. The analysis of the clinical data discovered potential biases due to age and weight. Therefore, statistical models were adequately corrected.

### 3.3 Correlations between metabolites and pathways and clinical outcomes

The metabolomic findings and correlations with clinical outcomes are summarized in Table 2.

**Table 2:** Metabolic findings and clinical outcomes.

	Main clinical outcomes									
	Relevant metabolic pathways and metabolites	Cardiac arrest, ECMO (extracorporeal membrane oxygenation), death in hospital or death within 30 days of surgery (inpatient or outpatient)	Composite Morbility/Mortality (met if any of the following occurred after surgery but before hospital discharge: death, ECMO, cardiac arrest, acute kidney injury, hepatic injury, increasing lactate levels)	Cardiac composite (extracorporeal membrane oxygenation, cardiac arrest, or increase in lactate level), hepatic injury and acute kidney injury.	Hipoxemia (Percent of time in the first 48 postoperative hours with an oxygen saturation below 70%)	AKI using the Kidney Disease Improving Global Outcomes (KDIGO) criteria with the neonatal modification for subjects ≤28 days of age.	ICU LOS	PICU-free days at 28 days	Other Predictive Scores	Neuropsychological examinations and an evaluation of the adaptive functioning witch includes the Raven Colored Matrices, the NEPSY-II, the Coding test of the WISC-IV, the visual-motor integration test and for adaptive functioning the Vineland Adaptive Behavior Scales (VABS-I).
Goncalo D. S. Correia, 2015		/	/	/	/	/	/	Lactate, creatine, creatinine, glucose, citrate, formate, 3-hydroxybutyrate, acetone, acetate, acetoacetate, alanine, valine, isoleucine, leucine, and threonine	(i) Pediatric Logistic Organ Dysfunction (PELOD) score (ii) RACHS-1 Early postoperative period (on PICU admission and at 6hr post surgery). Direct correlated: lactate, citrate, and to a lesser extent creatinine and alanine. Inverse correlated: ketone bodies (particularly acetoacetate) and creatine. (iii) Inotropic Score Direct correlated: alanine, citrate, and lactate. Inversed correlated: creatine.	/
Jesse A. Davidson, 2018		Aspartate (AUC, 0.87) and glutamate (AUC, 0.80) from alanine/aspartate/glutamate metabolism; methylnicotinamide (AUC, 0.84) and trigonelline (AUC, 0.80) from nicotinate/nicotinamide metabolism; kynurenic acid (AUC, 0.80) from tryptophan metabolism.	/	/	/	/	Decreased aspartate and glutamate and elevated methylnicotinamide, trigonelline and kynurenic acid.	/	/	/
Vedovelli, 2019	Relevant metabolic pathways and metabolites	/	/	/	/	/	/	No difference	/	Metabolites over-represented in the RED cluster: glucose, sucrose, acetate, formate, lactate, succinate, alpha-ketoglutarate, creatine, and urea meaning a higher levels of accumulation of citric acid cycle intermediates and glucose (possible switching to anaerobic metabolism)
Jesse A. Davidson, 2021		/	/	/	/	165 metabolites; 20 metabolites differed significantly between infants with and without AKI by their 24hr metabolic profile (AUC of >0.75). The combination of methylmalonic acid, kynurenic acid, 4-aminobenzoic acid (PABA), and pyrophosphate demonstrated the strongest differential ability (AUC = 0.92)	/	/	/	/
Heibel, 2022		/	193 metabolites examined of whitch 40 detected and quantified, 34 metabolites changed significantly: (t1) Ornithine,(t1) Phenylalanine, (t1) Methionine, (t3) Cystine, (t3) 4-Hydroxyproline, (t3) 3-Methoxytyrosine, (r21) Homoserine	Composite morbility/mortality: (t1) Ornithine, (t1) Phenylalanine, (t1) Methionine, (t3) Cystine, (t3) 4-Hydroxyproline, (t3) 3-Methoxytyrosine, (r21) Homoserine. Composite hepatic: (t1) Carnitine, (t1) Aspartic acid and (t3) Tyrosine. Composite lactate level: (t1) Methionine, (r31) Galactitol, (t3) Proline, (t3) Leucine.	**/**	(t1) Ornithine, (t1) Isoleucine, (t1) Aspartic acid, (t3) Alanine, (t3) Glutamic acid, (t3) 4-Hydroxyproline, (t3) Serine.	/	/	/	/
S.Frank, 2023					175 metabolites; possible association between postoperative tryptophan intermediates and hypoxemia burden.					
S.Frank, 2024	Relevant metabolic pathways and metabolites				9 metabolites: (i) at 2 h post-operation metabolites were associated with 48 h Low Sat%; (ii) at 24 h increased levels of ADMA and SDMA were associated with a greater post-operation hypoxemia, a one standard deviation increase in ADMA was associated with a 46.3% increase in expected 48 h Low Sat%		Arginine, citrulline, glutathione, ornithine, ADMA and SDMA were directly correlated to LOS: (i) a one standard deviation increase in citrulline concentration was associated with a 29.5% decrease in expected LOS; (ii) at 2 h post-Stage 2, decreased levels of four of the same metabolites were associated with longer LOS (arginine, citrulline, glutathione, ornithine); (iii) at 24 h post-Stage 2, persistently decreased levels of 3 metabolites showed association with longer LOS (citrulline, glutathione, ornithine).			

In Correia’s study, the analysis showed no significant differences in the primary outcome but a correlation between different metabolites and the secondary outcomes analysed [PICU-free days at 28 days, Pediatric Logistic Organ Dysfunction (PELOD) score, RACHS-1 score, Inotrope score and duration of ventilation].

Several metabolites were found to be positively correlated with *the number of PICU-free days at 28 days*: lactate, creatine, creatinine, glucose, citrate, formate, 3-hydroxybutyrate, acetone, acetate, acetoacetate, alanine, valine, isoleucine, leucine and threonine. Furthermore, the *Pediatric Logistic Organ Dysfunction (PELOD) score* and the *RACHS-1 score* on admission to the PICU and in the immediate postoperative phase (6 h after surgery) correlated directly with lactate, citrate, creatinine and alanine but inversely with ketone bodies (particularly acetoacetate) and creatine. The *Inotropic Score* was directly proportional to alanine, citrate, and lactate and inversely proportional to creatine.

In Davidson's first study, from 2018, a supervised dimension reduction technique (PLS-DA) analysis of the 24-hour metabolic fingerprint was able to discriminate between survivors and non-survivors. Moreover, it demonstrated reasonable discrimination between subjects with an upper vs. lower ICU length of stay of 50%, with modest performance in cross-validation.

The outcomes of *cardiac arrest*, *ECMO*, *in-hospital death or death within 30 days of* surgery (inpatient or outpatient) strongly correlated with aspartate (AUC 0.87) and glutamate (AUC 0. 80) from alanine/aspartate/glutamate metabolism, methylnicotinamide (AUC 0.84), and trigonelline (AUC 0.80) from nicotine/nicotinamide metabolism, and kynurenic acid (AUC 0.80) from tryptophan metabolism. Furthermore, the *ICU LOS* was related to a decrease in aspartate and glutamate and an increase in methylnicotinamide, trigonelline and kynurenic acid at various time points. The combination of 24-hour aspartate and methylnicotinamide had the greatest ability to predict this outcome.

In Davidson's second study, from 2021, the preoperative metabolic profile did not discriminate well between subjects who did or did not develop AKI in the first 72 h after surgery. However, the 24-hour postoperative metabolic profile provided moderate differences between subjects without AKI and those who subsequently developed moderate to severe AKI. The metabolic profiles of stage 1 AKI patients segregated into two groups overlapping either with subjects demonstrating no AKI or those demonstrating moderate to severe AKI. The absolute peak postoperative creatinine was significantly greater in the group of subjects with a severe metabolic phenotype; this group included the subjects who later progressed to stage 3 AKI by postoperative day 7.

Several metabolites and related metabolic pathways were found to be correlated with *AKI,* including the purine pathway (allantoate, uric acid, xanthine), cysteine/methionine/taurine pathway (serine, cystathionine, s-methyl-5-thioadenosine, s-adenosyl-L-homocysteine, taurine), kynurenine/nicotinamide pathway (kynurenic acid, 1-methylnicotinamide, trigonelline, aspartate), pyrimidine pathway (methylmalonic acid, uridine), amino acid acetylation pathway (N-acetyl-alanine, acetyl-L-lysine), and arginine pathway [putrescine, creatinine; other 4-aminobenzoic acid (PABA) and PPi]). In total, 20 metabolites differed significantly between infants with and without AKI according to their 24-hr metabolic profile (AUC>0.75). The combination of methylmalonic acid, kynurenic acid, 4-aminobenzoic acid (PABA), and pyrophosphate had the strongest effect (AUC = 0.92).

In Vedovelli's study from 2019, clustering in the “RED” and “BLACK” profiles successfully predicted late neurological dysfunction but failed to predict other clinical outcomes (i.e., PICU-free days at 28 days, ICU stay, total length of stay and duration of mechanical ventilation).

Several metabolites, such as glucose, sucrose, acetate, formate, lactate, succinate, alpha-ketoglutarate, creatine and urea, were found to be overrepresented in the RED cluster. This could mean a greater accumulation of citric acid cycle intermediates and glucose, possibly resulting in a switch to anaerobic metabolism. Considering the clinical index (CI), 11/12 children in the RED metabolic cluster and 2/7 in the BLACK cluster met the criteria for neuropsychological dysfunctions (PPV 92%, NPV 71%, specificity 83%, sensitivity 85% and overall accuracy 84%). The H-NMR spectra predicted the infants’ neurodevelopment in a better fashion than did their anatomical and/or clinical classification.

In 2022, Heibel's study revealed that 55 patients (37%) experienced a composite morbidity/mortality outcome *(defined as occurrence after surgery but before discharge, ECMO, cardiac arrest, AKI, hepatic injury or increasing lactate levels)*, 34 patients (23%) experienced a cardiac composite outcome (*defined as the presence of ECMO, cardiac arrest or increased lactate levels*), 66 patients (44%) experienced AKI, 29 patients (19%) experienced elevated lactate levels, and 38 patients (26%) experienced hepatic injury. The addition of PC1 significantly improved the clinical model performance for the composite morbidity/mortality outcome, composite cardiac outcome and hepatic injury, particularly PC1 + PC3 for AKI and PC1 + PC6 for lactate level.

Of the 193 metabolites examined, 40 were detected and quantified, 34 of which were correlated with the *composite morbidity/mortality outcome* and cardiac *composite outcome*, *hepatic injury*, and *acute kidney injury*. The most relevant ones for morbidity/mortality were (t1) ornithine, (t1) phenylalanine, (t1) methionine, (t3) cystine, (t3) 4-hydroxyproline, (t3) 3-methoxytyrosine, and (r21) homoserine; for the composite liver, (t1) carnitine, (t1) aspartic acid and (t3) tyrosine were used. The composite lactate levels were as follows: (t1) methionine, (r31) galactitol, (t3) proline, and (t3) leucine. The AKI levels were as follows: (t1) ornithine, (t1) isoleucine, (t1) aspartic acid, (t3) alanine, (t3) glutamic acid, (t3) 4-hydroxyproline, and (t3) serine. PC1 alone was significantly associated with composite morbidity/mortality, and 14 of the 20 metabolites within this group were detected preoperatively.

In S. Frank's studies from 2023 to 2024, 71 of 174 individual metabolites and 3 of 39 assessed pathways differed significantly between samples obtained before and 2 h after surgery. Metabolites related to arginine metabolism and biosynthesis, tryptophan metabolism, glycine metabolism, and pyrimidine metabolism were able to distinguish between the two groups with a sensitivity of 97.9% and specificity of 96.2%. Additionally, these metabolites could be used to divide the SVHD population into those with elevated PVRi (>3 WU/m^2^) and those with normal PVRi (< 3 WU/m^2^). In the second study, postoperative outcome analysis revealed that eight of the nine measured metabolites changed during the perioperative period. Several metabolites were associated with the considered outcomes: (i) At 2 h post-operation, certain metabolites were associated with 48-hour low saturation percentages (low Sat%), while at 24 h, increased levels of Asymmetric Dimethyl Arginine (ADMA) and Symmetric Dimethyl Arginine (SDMA) were linked to greater postoperative hypoxemia. A one standard deviation increase in ADMA was associated with a 46.3% increase in the expected 48-hour low Sat%. (ii) Six of the nine arginine pathway metabolites (arginine, citrulline, glutathione, ornithine, ADMA and SDMA) were associated with increased post-stage 2 length of stay. An increase of one standard deviation in the citrulline concentration was associated with a 29.5% decrease in the expected LOS. At 2 h post-stage 2, decreased levels of four metabolites (arginine, citrulline, glutathione, ornithine) were further associated with a longer LOS; at 24 h post-stage 2, persistently decreased levels of three metabolites (citrulline, glutathione, ornithine) again showed a significant association with a longer LOS. (iii) Multiple logistic regression demonstrated an association between decreased citrulline levels at both 24 h and 48 h and increased odds of discharge after PDE5 inhibitor therapy.

#### 3.3.1 Quality of evidence

Despite the promising findings, the quality of evidence from the included studies is limited by several factors. The heterogeneity in study designs, patient characteristics, and underlying diagnoses, along with small sample sizes, variations in metabolomic analysis techniques, and differences in data analysis methods, present significant challenges to the generalizability and robustness of the conclusions. Additionally, given the incidence of CHD, the majority of studies included a population of a wide age range, although the metabolic pathways in newborns are not so developed than those in infants and older children (18).

The results of the included studies should be considered exploratory.

First of all, the observational nature of the considered studies limits the ability to infer causality between metabolic alterations and clinical outcomes.

Second, data analysis was not adequately reported in most of the selected studies, lacking some important information about model optimization and validation. Moreover, the absence of over-fitting in multivariate modelling was not proved. Despite the efforts of the Metabolomics Standards Initiative in suggesting guidelines for reporting the metabolomics data, experimental analysis and statistical data analysis (19), most of his recommendations were not applied, limiting the generalizability and the quality of the findings.

Moreover, in most of the selected exploratory studies, the experimental design was not explicitly included in data modelling. Statistical tools, mainly PCA and PLS-based, were applied to investigate complex scenarios, where confounding effects may generate biases. The use of mixed-effect models controlling false discovery rate or more advanced multivariate tools such as PERMANOVA (20) may improve the quality of data analysis in future studies.

## 4 Discussion

Despite significant improvements over the past 30 years, surgery for congenital heart diseases still carries a high burden of morbidity and mortality, especially in high-risk populations such as neonates (1, 2). Several risk models and scores have been proposed to predict patients with the most unfavorable outcomes, but these models mainly incorporate clinical factors. Metabolomics can provide a close picture of ongoing physiological and deranged pathways in a biological system, potentially revealing new predictive biomarkers and therapeutic targets. This omic science has already been applied in the field of CHD and has already proven its feasibility of non-invasive screening for congenital heart defects using a serum metabolomics approach (21–24). A study published by Bahado-Singh in 2014 on metabolomic biomarkers in maternal first-trimester blood revealed significant differences in sphingomyelin, acylcarnitine, and other metabolite levels associated with fetuses affected by CHD: predictive algorithms for CHD showed an excellent (>0.9) sensitivity and specificity (25). Another study using untargeted metabolomic analysis revealed that amniotic fluid uric acid levels had moderate predictive power in identifying cases of CHD (23).

Currently, an increasing number of studies are focusing also on the potential prognostic role of metabolomic profiling for understanding the complex biochemical changes in infants undergoing cardiopulmonary bypass (CPB) for congenital heart disease (CHD). The application of metabolomic analysis for predicting post-surgical outcomes has already been demonstrated in adult patients (7, 8). The studies included in this review consistently demonstrate that metabolomic signatures can be correlated with critical clinical outcomes after surgery also in a paediatric field, offering new insights into physio-pathological pathways and patient-specific responses.

The majority of the studies included in our review applied a targeted approach (12, 13, 15–17) and utilized serum samples (5, 12, 13, 15–17).

Correia et al. (5) reported that certain metabolites, such as lactate, creatine, and various amino acids, collected as early as admission to the PICU correlated with Pediatric Logistic Organ Dysfunction (PELOD) scores and other postoperative outcomes. In general, ketone bodies seem to correlate with better surgical outcomes, whereas citrate, lactate, alanine, and a higher creatinine-to-creatine ratio are inversely correlated. These results suggest that metabolic disturbances are closely linked to organ dysfunction and recovery trajectories in the immediate postoperative period and align with previously published research in the literature (26). The increased secretion of catecholamines, cortisol, and glucagon in critical illness patients promotes lipolysis and the generation of ketone bodies. During this process, triglycerides are broken down into fatty acids and glycerol, which is a gluconeogenic substrate. Therefore, high levels of glucagon and low levels of insulin further support the oxidation of free fatty acids to acyl CoA, which is then converted in the liver into ketone bodies (β-hydroxybutyrate, acetoacetate, and acetone) to serve as a water-soluble energy source (27).

Similarly, Davidson et al. (12, 13) showed a strong correlation between early postoperative metabolic profiles involving amino acid, purine and pyrimidine pathways (aspartate, glutamate, etc.) and critical outcomes such as the ICU LOS. This further supports the notion that specific metabolic pathways are disrupted very early in severe cases, potentially serving as early biomarkers for adverse outcomes.

The two main pathways involved seemed to be nicotinamide and aspartate metabolism. Differences in these pathways were noted in non-survivors and those with longer ICU stays, highlighting their potential postoperative biological relevance. Notably, metabolite pairs such as aspartate/glutamate and methylnicotinamide/trigonelline were key indicators of variations between survivors and non-survivors, as well as ICU length of stay.

Aspartate and glutamate are crucial for multiple metabolic pathways, including nitrogen metabolism and amino acid metabolism. Their maintenance or increase post-surgery is normal (28); indeed, that failure to do so could contribute to poor outcomes in neonates and infants. In contrast, methylnicotinamide and trigonelline have more recently been shown to have anti-inflammatory effects and potential responses to poor cardiac output; thus, excessive production or reduced clearance of these metabolites might contribute to vasoplegia (29). In regard to AKI, the authors observed an association between lower levels of uric acid and xanthine and patients who developed moderate/severe AKI. A possible reason for these findings is the increase in postoperative oxidative stress, which leads to the depletion of uric acid and to organ damage considering its important antioxidant role.

Kynurenic acid and nicotinamide were also found to be associated with AKI. Elevated levels of kynurenic acid were already found to be associated with increased ICU stay duration and cardiovascular failure at the time of the first study. In the second study, the same metabolites were found to be directly related to AKI possibly due to their role as NMDA receptor antagonists in the kidney (30). The same physio-pathological mechanism could also explain why nicotinamide, which plays an important role in NAD production, seems crucial for kidney health. In patients with experimental AKI, NAD depletion is observed due to increased consumption and reduced *de novo* synthesis (31). Elevated postoperative levels of metabolites such as methyl nicotinamide and trigonelline have been linked to a greater risk of AKI.

Ultimately, the elevated levels of cystathionine and decreased levels of taurine may indicate, in the first case, alterations in sulfur metabolism with potential effects on decreased intracellular antioxidants, while in the second case, increased consumption of this amino acid. Both lead to an increased oxidative stress response during AKI (32).

In S. Frank's studies (16, 17), it was proven that interstage infants with SVHD have significantly altered arginine-nitric oxide metabolism, including a deficiency of the pathway entry-point molecule arginine. These affected infants experience further deficiencies in multiple pathway intermediates in the immediate post-stage 2 period, with changes persisting through the first 48 postoperative hours. After controlling for clinical covariates and classic catheterization-derived predictors of Stage 2 readiness, both lower preoperative and postoperative circulating metabolite levels were associated with a longer postoperative stage 2 length of stay. Additionally, an increased post-stage 2 ADMA concentration was associated with a greater post-operative hypoxemia burden, while decreased citrulline levels were associated with increased odds of discharge on targeted pulmonary hypertension therapy.

To explain these results, the authors propose that alterations in arginine metabolism are critical because they affect the production of nitric oxide (NO), a vital vasodilator involved in the regulation of vascular tone and pulmonary blood flow. Decreased levels of arginine and citrulline reduce the synthesis of NO, thereby worsening vascular function and increasing vascular resistance, which could lead to greater postoperative complications and extended hospital stays (33). Furthermore, the increased concentration of ADMA, a known inhibitor of nitric oxide synthase, further exacerbates these effects by reducing NO production, contributing to the post-operative hypoxemia observed (34, 35). This dysregulation of arginine metabolism and NO synthesis highlights potential targets for therapeutic intervention, aiming to optimize NO levels to improve outcomes in infants undergoing stage 2 palliation.

The same significant disruptions in the arginine biosynthesis and aminoacyl-tRNA biosynthesis pathways was found also in the metabolic profiling approach used by Heibel et al. (15) to predict composite morbidity and mortality outcomes. The authors suggest that these disruptions may lead to reduced synthesis of nitric oxide, a critical regulator of vascular tone and blood pressure, which could exacerbate perioperative vascular instability and contribute to adverse outcomes such as increased morbidity and mortality.

Finally, Vedovelli et al.’s (14) identification of metabolic clusters (“RED” and “BLACK”) that predict neurological dysfunction also emphasizes the utility of metabolomics in prognosticating long-term neurodevelopmental outcomes.

As underlined, limited research has been published in this field to date, with each study focusing on different aspects such as the patient population studied, the sample size, patient characteristics, and particularly the type of specimens analysed. Additionally, there is considerable heterogeneity in the metabolomics analysis techniques used, the statistical methods applied, the outcomes targeted, and the postoperative time points at which these outcomes are assessed.

In particular, it must be taken into account that the choice of the mass spectrometry technique can greatly influence the metabolites identified and likely accounts for some of the differences in the findings. All metabolomics investigations, whether employing targeted or untargeted methods, are inherently biased, despite the holistic aims of metabolomics as a discipline. This bias arises because the analytical technique used to measure the metabolite content of a biological sample defines the chemical space considered, which in turn determines the biological framework within which the behavior of the system under investigation is interpreted. In practice, different analytical techniques may detect different metabolites in the same set of samples, potentially leading to divergent biological interpretations for the same process. This is not surprising, given the complexity of biological systems and the extensive range of metabolic perturbations that can occur, often affecting numerous pathways. Moreover, no single analytical platform currently exists that is capable of capturing the entire metabolome. The studies reviewed here employed different analytical platforms. Correia et al. and Vedovelli et al., for example, used 1D-HNMR-based metabolomics. Specifically, Correia et al. quantified a panel of 15 serum metabolites selected based on existing literature on the metabolic response to critical illness, while Vedovelli et al. employed an untargeted approach, considering the entire spectral region without pre-selecting specific NMR signals. In contrast, other studies utilized targeted approaches based on liquid chromatography-tandem mass spectrometry. With the exception of the confirmatory study by Frank et al. (2024), no clear rationale for the choice of metabolite panels was provided. Even with the use of similar technologies, the implemented metabolomics assays varied considerably. Unfortunately, only Hiebel et al. and Frank et al. (2024) reported the complete list of metabolites included in their panels, while the other studies listed only the metabolites deemed relevant after data analysis. Consequently, it was not possible to determine whether the same chemical space was sampled across these studies. Additionally, no untargeted approaches based on mass spectrometry were applied, despite the exploratory nature of most of the studies. For improved clarity and reproducibility, we recommend that future studies report the complete list of metabolites included in their panels, provide a detailed description of the analytical procedures along with robustness tests and performance metrics, and justify the selection of the targeted panel. Furthermore, in exploratory studies, untargeted approaches, which cover a broader chemical space, should be preferred to generate hypotheses that can be subsequently tested using targeted methods.

Naturally, the aforementioned heterogeneity complicates the comparability of the results. Moreover, patient medication was not accounted for, which can significantly influence metabolic status, especially in the ICU setting.

Despite these variations, it is noteworthy that a specific subset of metabolites and key metabolic pathways consistently appear to be altered across multiple studies. Among others *those related to* ketone body pathway as well as the tryptophan/kynurenine/nicotinamide pathway, and the arginine pathway*, were consistently associated with post-cardiac surgery outcomes, including neurological impairment and acute kidney injury (AKI)*, all of which suggest disruptions in energy metabolism, stress response, and endothelial function (12, 13, 15, 17).

### 4.1 Clinical implications

Ultimately, the results of this review confirm the encouraging applicability of metabolomics in paediatric patients with congenital heart disease. In addition, this study offers interesting insights into possible therapeutic targets in this fragile population. For example, in Frank's studies (16, 17), arginine-NO metabolism was found to be a promising therapeutic target for the subset of SVHD patients with impaired pathway activity. Evaluating whether patient-level arginine metabolism mapping could be used as a strategy to identify SVHD patients most likely to benefit from these interventions and thereby inform personalized medicine and biomarker-directed approaches for future therapeutic trials is an exciting area for future studies. Targeted interventions to modulate specific metabolic pathways (e.g., through nutritional support or pharmacological agents) could be employed to mitigate the identified risks. In this regard, in Correia's study (5), changes in diet rapidly and significantly affected the metabolic profile. Compared with healthy children, children with congenital heart disease exhibit early and progressive decreases in their growth trajectory and it is also known that surgery, bypass procedures, and the burden of cardiac failure and chronic disease result in significant metabolic and nutritional stress (36, 37). For these reasons, the authors concluded that it would be interesting to compare the metabolic profile of this cohort of patients who received standard nutritional care pre- and postoperatively with that of well-nourished children. The ability to identify high-risk infants through preoperative and immediate postoperative metabolic profiling could significantly change perioperative care strategies and integrating metabolomic data with existing clinical risk models could enhance their predictive power, leading to more precise and individualized patient management plans.

In conclusion, the applicability of these findings to pediatric populations undergoing cardiopulmonary bypass (CPB) suggests that metabolomic profiling could fill a critical gap in early diagnosis and intervention, enabling tailored perioperative care to mitigate risks.

### 4.2 Future directions

Future research should aim to apply also more robust statistical tools to reduce the risk of confounding biases, and to report metabolomics data, experimental analysis and statistical data analysis according to Metabolomics Standards Initiative (20) in order to better elucidate the mechanistic pathways linking metabolic disturbances to clinical outcomes in infants undergoing CPB. Longitudinal studies with a strict selection of population tracking metabolic changes from preoperative stages through long-term follow-up would be particularly valuable. Additionally, exploring the integration of metabolomics with other omics approaches (e.g., genomics, proteomics) could provide a more comprehensive understanding of the biological processes involved and identify novel therapeutic targets. Efforts should also be made to develop and validate metabolomic biomarkers for routine clinical use, ensuring that the insights gained from research translate into tangible benefits for patient care.

## 5 Conclusions

In conclusion, metabolomic profiling holds significant promise for improving the care of infants undergoing CPB for CHD. By providing deeper insights into the metabolic alterations associated with surgical stress and recovery, metabolomics can help identify high-risk patients, guide perioperative management, and ultimately assess clinical outcomes. Continued research and collaboration in this field are essential to fully realize the potential of metabolomics in pediatric cardiac surgery.

## Data Availability

The original contributions presented in the study are included in the article/Supplementary Material, further inquiries can be directed to the corresponding author.
